# Value of Early Diagnosis and Treatment of Amyloidosis: A Pilot Study of Synovial Biopsy During Carpal Tunnel Release

**DOI:** 10.1016/j.jhsg.2025.100779

**Published:** 2025-07-23

**Authors:** Trevor Ruesch, Nevil Khurana, Logan Hansen, Katiya Barkho, Julia Malewicz, Benjamin Brennan, Charles S. Day

**Affiliations:** ∗ Wayne State University School of Medicine, Detroit, MI; † Department of Orthopedic Surgery, Henry Ford Health, Detroit, MI

**Keywords:** Amyloidosis, Carpal tunnel syndrome, Synovial biopsy, Tafamadis, Value

## Abstract

**Purpose:**

The purpose of this study is to calculate the value of early diagnosis and treatment of transthyretin amyloidosis with tafamadis prior to the development of the symptoms of heart failure. In this pilot study of 51 patients, we present the validation of a published algorithm for the early identification of patients at risk for amyloidosis via tenosynovial biopsy during carpal tunnel release. In addition, by integrating clinical data from this pilot study with published predictive models, we aim to calculate the value of routine screening biopsies for transthyretin amyloidosis.

**Methods:**

Patients presenting for carpal tunnel release surgery had a tenosynovial biopsy collected at the time of surgery. Cost information was gathered from hospital records. In conjunction with published models, five incremental cost effectiveness ratio equations were generated to assess the value of these screening biopsies.

**Results:**

Of the 51 biopsied patients, six tested positive for amyloid, and one was started on tafamadis, a disease-modifying medication. Early diagnosis and treatment of patients with New York Heart Association class I (NYHA I) heart failure as opposed to NYHA IV results at a cost of $166,691.49 USD per quality adjusted life year (QALY). When treatment is initiated at NYHA class II stage compared with NYHA class IV, there is a cost of $155,977.22/QALY. For treatment at NYHA class III compared with NYHA class IV, the cost is $75,333.28/QALY.

**Conclusions:**

This study validates the utility of previous criteria in identifying patients at high risk for systemic amyloidosis earlier in the disease course. Using the commonly accepted willingness to pay threshold of $50,000/QALY, early initiation of tafamadis does not represent a cost effective intervention. Routine biopsy of patients is not cost effective with the current cost of therapy and positivity rates of amyloidosis screening.

**Type of study/level of evidence:**

Prognostic IB.

Amyloidosis is caused by the accumulation of misfolded protein fibrils, resulting in end-organ toxicity.^1^ These insoluble fibrils can accumulate in a variety of tissues across the body, commonly impacting the myocardium.^2^^,^^3^ Accumulation within the myocardium leads to oxidative stress and fibrosis, progressing to restrictive cardiomyopathy and heart failure with preserved ejection fraction.^3^ The nonspecific clinical presentation of amyloidosis leads to delays in diagnosis and treatment, contributing to a high mortality rate.^4^ In the landmark Transthyretin Amyloidosis Cardiomyopathy Clinical Trial (ATTR-ACT), all-cause mortality at 30 months after diagnosis for transthyretin amyloidosis (ATTR) without disease-modifying treatment was 42.9%, with untreated survival after diagnosis ranging from 31 to 69 months.^5^^,^^6^ Diagnosis often requires multiple diagnostic tests, ranging from echocardiography to cardiac magnetic resonance imaging (MRI) and radionuclide scanning.^7^^,^^8^ There is still opportunity for more frequent intervention even after diagnosis, with a prior study showing only 58.8% of patients diagnosed with transthyretin amyloid cardiomyopathy being prescribed disease-modifying therapy.^9^

Early diagnosis in milder stages of heart failure has become even more imperative with the advent of modern disease-modifying treatments. Tafamadis is a medication which can prolong the period during which patients remain in earlier stages of heart failure, considerably increasing the incremental quality adjusted life-years (QALY) gained from treatment.^5^ Tafamadis functions by preventing the misfolding and aggregation of transthyretin proteins into the amyloid fibrils that cause the symptoms of ATTR. Patients diagnosed with ATTR and treated with tafamadis had a 30-month all-cause mortality rate of 29.5% compared to 42.9% in untreated patients, an absolute risk reduction of 13.4%.^10^ Continuous treatment with tafamidis over a five-year period was associated with an improved five-year survival rate of 53.2%, whereas patients who delayed treatment 30 months after diagnosis had a five-year survival rate of only 32.4%.^6^^,^^11^ Despite its demonstrated benefits in reducing morbidity and mortality as well as improving quality of life, tafamadis remains largely cost-prohibitive, with an estimated annual cost of approximately 225,000 USD per patient.^12^ Given the currently high price of the medication, optimizing treatment timing is vital to the health care system.^12^ Furthermore, patients with ATTR typically undergo carpal tunnel release (CTR) 5.1 years before their eventual diagnosis.^13^ This early musculoskeletal manifestation represents a window of opportunity for earlier diagnosis, as amyloid deposits accumulate in the transverse carpal ligament and tenosynovium–tissues that are readily accessible for biopsy during CTR.^14^, ^15^, ^16^, ^17^

The purpose of this study is to calculate the value of early diagnosis and treatment of transthyretin amyloidosis with tafamadis prior to the development of the symptoms of heart failure. This assessment is based on data collected in a pilot study of biopsies performed during CTR, supplemented by published predictive models on disease-modifying treatment. We hypothesize that the findings of this pilot study will be in alignment with the current body of literature, which estimates a 10% to 15% positivity rate for ATTR using existing screening algorithms and that earlier diagnosis afforded by biopsy at the time of CTR results in a more cost effective treatment of ATTR.

## Materials and Methods

**Figure 1:**
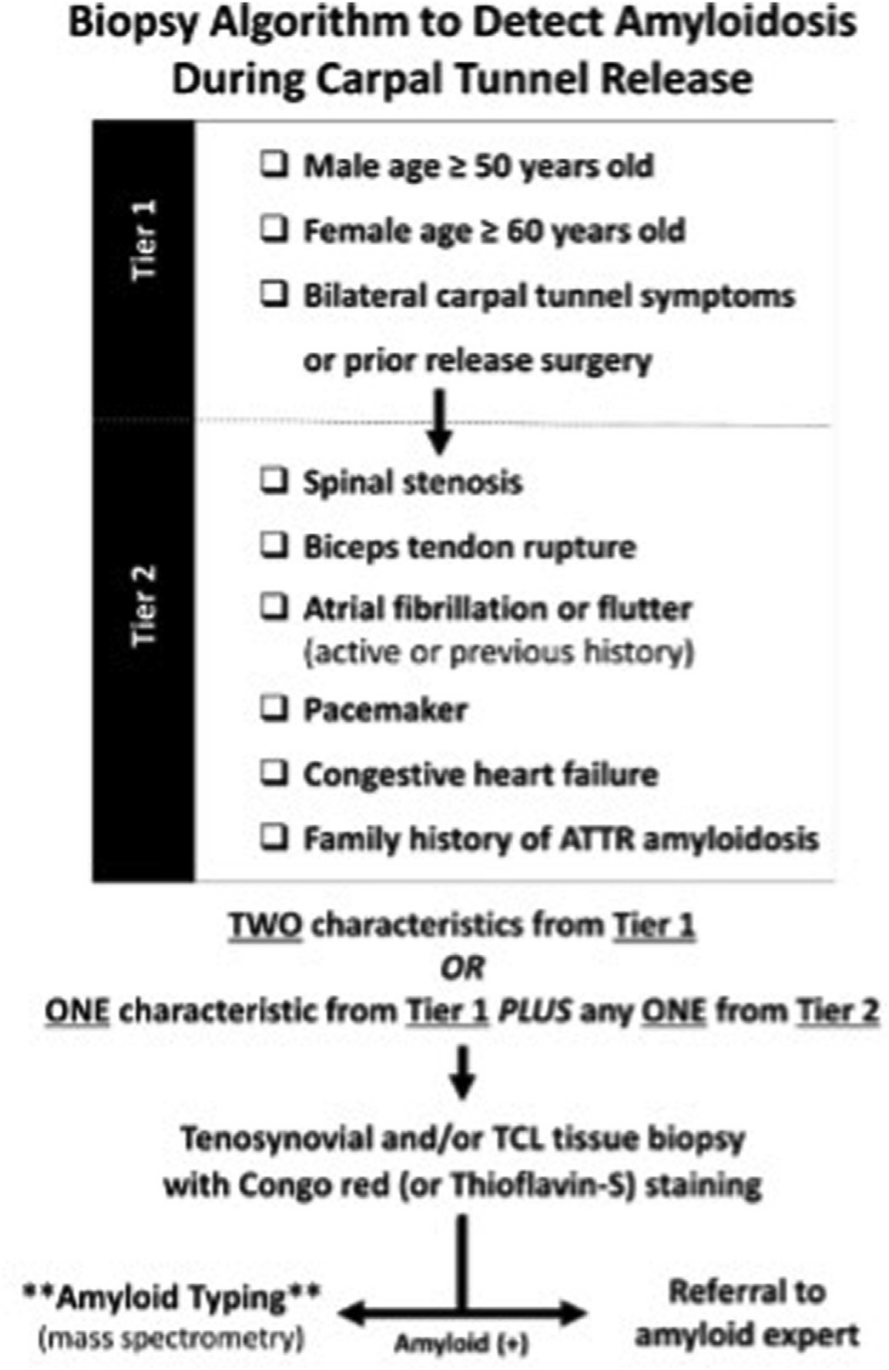
Screening Algorithm established by Sperry et al^18^ used for inclusion criteria.

All patients scheduled to undergo open or endoscopic elective CTR with the principal investigator were screened for participation. Screening occurred at the beginning of each week from the electronic medical record according to the screening criteria described by Sperry et al^18^ and outlined in the Figure 1. Study participation was offered to patients who met the inclusion criteria. This was defined as two characteristics from the Tier 1 criteria, or one characteristic from the Tier 1 criteria and one characteristic from the Tier 2 criteria. Tier 1 criteria were defined as men aged ≥ 50 years or women aged ≥ 60 years and bilateral carpal tunnel symptoms or prior release surgery. Tier 2 criteria were defined as a diagnosis of spinal stenosis, biceps tendon rupture, atrial fibrillation or flutter (active or previous history); presence of a pacemaker; diagnosis of congestive heart failure; or a family history of amyloidosis. Patients were excluded from participation if they were under the age of 18, belonged to a vulnerable population, or had a previous diagnosis of amyloidosis. Informed consent was obtained before surgery on the day of the scheduled operation. All carpal tunnel surgeries were performed by the principal investigator via an endoscopic system.^19^ The procedure was performed under a regional block with conscious sedation. After release of the transverse carpal ligament, a biopsy was obtained from either a portion of the transverse carpal ligament or the tendon sheath of the flexor tendons within the carpal tunnel. This is consistent with previous literature equating the two sites of biopsy.^20^ The biopsy specimens were immediately stored in 10% formalin, at 15–20× the volume of the surgical specimen. After the conclusion of the case, specimens were transported to a central pathology laboratory, stained with Congo red dye, and interpreted by board-certified pathologists.^21^ At the scheduled after surgery follow-up visit, biopsy results were reviewed with the patient. Any patient with a biopsy positive for amyloid was referred for further evaluation with a board-certified cardiologist in our hospital system specializing in care of amyloidosis.

**Figure 2:**
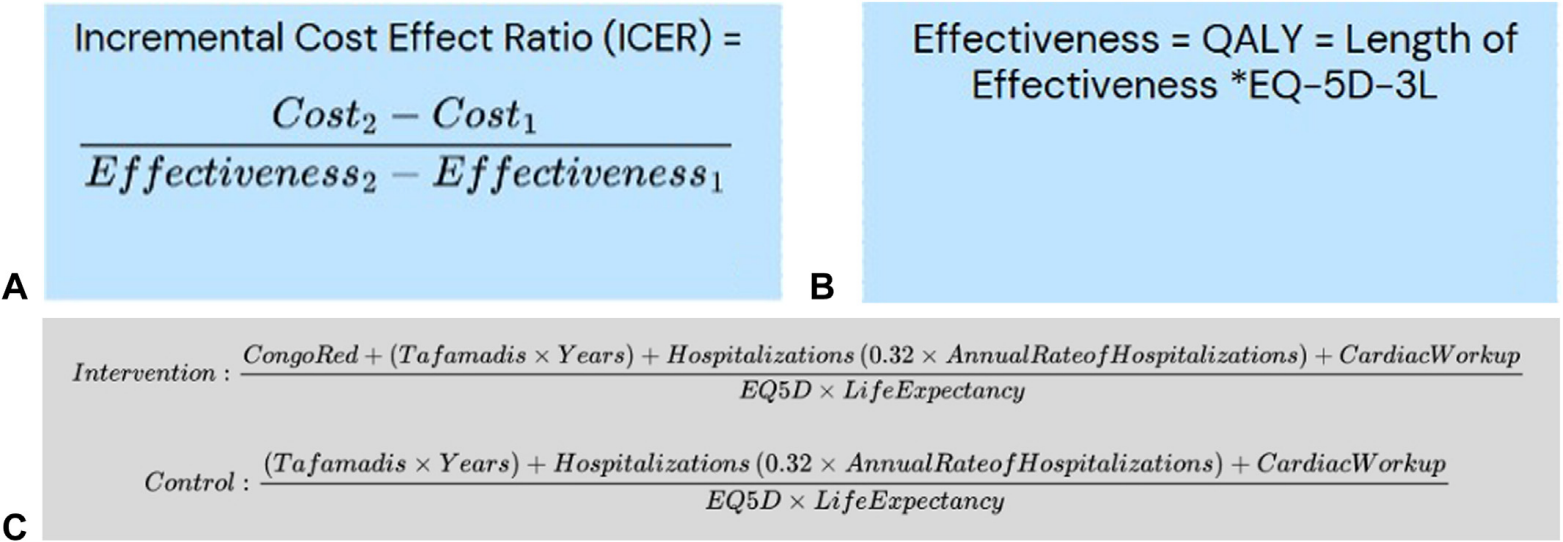
**A** Description of the components of an ICER equation. **B** Breakdown of the components of effectiveness, which is included in denominator of the ICER equation. **C** ICER equations used for value calculation. Congo red, cost associated with pathology for Congo red stain; Tafamadis, wholesale acquisition price aka “list” price per year multiplied by the estimated life expectancy of each stage of heart failure; Hospitalizations, cost associated with hospitalizations stratified by NYHA heart failure classification; CardiacWorkup, costs associated incurred by cardiac MRI, nuclear scintigraphy, cardiac SPECT, echocardiogram, and monoclonal antibody screen; EQ5D, associated utility modifier stratified by NYHA classification.

In addition to this prospective pilot study, this study investigated the potential value of early screening and diagnosis of amyloidosis. A standard incremental cost effectiveness ratio (ICER) equation (Fig. 2A) was created. The cost for each respective New York Heart Association (NYHA) classification was calculated via the summation of the annual cost of tafamadis, the cost per hospitalization, the cost associated with pathologic staining, and finally the costs of cardiac work-up.^12^^,^^22^^,^^23^ The cost of tafamadis was defined as the annual list price of tafamadis multiplied by the life expectancy of each stage of heart failure.^12^^,^^22^ The costs per hospitalization were gathered in 2013 euros, converted to USD via the average exchange rate in 2013 of 1.33, and then multiplied by the annual rate of hospitalizations and the relative risk reduction of tafamadis therapy.^10^^,^^22^^,^^23^ The cost of pathology was calculated via the average hospital charge data from the collected sample of patients. Finally, the cost of cardiac work-up included the average costs incurred from cardiac MRI, cardiac nuclear scintigraphy, cardiac single photon emission computed tomography scan, echocardiography, and serum monoclonal protein screening from our patient population. For the QALY component in the denominator of the ICER equation, a EuroQol 5-Dimension 3-Level (EQ-5D-3L) score was used (Fig. 2B). This EQ-5D-3L score was obtained by correlating NYHA scores for heart failure classes I-IV with a Kansas City Cardiomyopathy Questionnaire (KCCQ) overall score (OS) as outlined by Greene et al^24^, followed by performing a crosswalk calculation described by Thomas et al^25^ on the KCCQ score to obtain the final EQ-5D-3L score (Table 1). Four ICER equations were calculated comparing interventions with tafamadis in different NYHA classes of heart failure. These were class I compared to class IV, class I compared to class III, and class I compared to class II. Additionally, to account for a hypothetical treatment initiation prior to the development of any signs or symptoms of heart failure, an additional ICER equation comparing treatment starting in an asymptomatic theoretical NYHA class 0 and class IV was generated. Patients in the class 0 category are assumed to have no annual hospitalizations because of heart failure and as such would accrue no subsequent hospital costs. The assumption of no cardiac symptoms equating to an EQ-5D of 1, average life expectancy in agreement with current United States census data and the average age of CTR surgery were terms used for the denominator of the ICER equation.^26^^,^^27^ The ICER denotes the predicted cost/QALY differential. A willingness to pay threshold of $50,000/QALY was used for value assessment.^28^^,^^29^

**Table 1:** Results of Crosswalk Calculation of KCCQ to the Corresponding EQ-5D Stratified by NYHA Classification

NYHA Classification	KCCQ-OS	EQ-5D
1	85	0.83
2	70	0.80
3	55	0.73
4	45	0.68

EQ, EuroQol; KCCQ-OS, Kansas City Cardiomyopathy Questionnaire overall summary score.

The sample equation for comparing tafamadis initiation in NYHA class I heart failure and initiation in NYHA class IV heart failure is depicted in the Figure 2C. The values from the control group were subtracted from the values of the intervention group to determine final ICER values. Control groups for the ICER equations are defined as having no biopsy and initiation of tafamadis at NYHA class II or greater. The proposed value, as calculated from each ICER equation, was compared to the overall cost of the negative biopsies to determine overall cost effectiveness. This study was approved by the institutional review board of our institution (protocol #6239-01) and registered through ClinicalTrials.gov, ID number: NCT05793320.

## Results

In total, 51 patients who met the inclusion criteria were recruited from a multicenter tertiary care hospital system from September 2023 until December 2024. Of these, 11.7% (n = 6) of the patients had positive biopsy results, and 88.3% (n = 45) patients who had negative biopsies. Of the 51 patients included, 84% (n = 43) had no previous diagnosis of heart failure (Table 2). The six patients with positive biopsies were referred to high risk cardiology for follow-up. Of these patients, 83% (n = 5) followed up with cardiology. Of the patients that were seen by the cardiologist, 40% (n = 2) underwent nuclear medicine scanning, and 20% (n = 1) started tafamadis therapy.

**Table 2:** Descriptive Statistics for Patients Included in the Study Stratified by Biopsy Positivity

Biopsy Result (N)	Positive (6)	Negative (45)
Age (y)	74.1 ± 10.1	69.98 ± 8.68
Male Sex (%N)	67 (4)	38 (17)
Bilateral carpal tunnel (%N)	100 (6)	98 (44)
Heart failure (%N)	33 (2)	13 (6)
Pacemaker (%N)	0 (0)	2 (1)
Lumbar spinal stenosis (%N)	33 (2)	31 (14)
Atrial fibrillation (%N)	16 (1)	20 (9)
Biceps rupture (%N)	0 (0)	9 (4)

The ICER differential for tafamadis treatment comparing class IV NYHA heart failure versus class I NYHA heart failure was $166,691.49 USD/QALY. The ICER differential for tafamadis treatment comparing class III NYHA heart failure versus class I NYHA failure was $155,977.22 USD/QALY. The ICER differential for tafamadis treatment comparing class I NYHA heart failure versus class I NYHA heart failure was $75,333.28 USD/QALY. Finally, the ICER differential for hypothetical class 0 patients, who had no signs or symptoms of heart failure and therefore could not be classified as NYHA class I, compared with class IV NYHA was $202.417.74 USD/QALY. The values used for each variable included in the ICER equation are listed in Tables 3 and 4. The total cost of Congo red staining for the patient cohort with a negative biopsy was $10,176.30 based on a mean cost for associated pathology per biopsy of $226.14 (n = 45). To biopsy all 51 patients, the associated pathology costs were $11,533.14.

**Table 3:** Variables Used in the ICER Equations for the Calculation of Value

	Hospitalizations Because of Heart Failure Exacerbation	EQ-5D Stratified by NYHA Classification	Incremental Life Extension Because of Tafamadis Initiation (y)	Mean Hospitalization Costs per NYHA Classification (USD)
NYHA Class I	14.2%	0.83	1.67	3,009.79
NYHA Class II	17.1%	0.80	1.55	3,468.64
NYHA Class III	32.5%	0.73	0.64	4,684.26
NYHA Class IV	33.3%	0.68	0.02	8,630.37

EQ-5D, EuroQol; USD, United States dollars.

**Table 4:** Variables Used in the ICER Equation Continued

Cost of tafamadis per year (USD)	225,000
Cost of Congo red staining (USD)	226.14
Relative risk reduction for tafamadis and hospitalizations	0.32
Average cost for cardiac work-up (USD)	19,880.25
Life expectancy while on tafamadis (y)	5.58

## Discussion

Carpal tunnel release is a high-volume procedure performed by hand surgeons, which would present a considerable opportunity to intervene for patients at high risk of amyloidosis. Biopsy of these patients during their surgery can provide an early window for treatment. Our positive biopsy rate, at 11.7%, was in line with those of Sperry et al^18^ at 10.2%, as well as a recent systematic review by Donnelly et al^30^ that estimated a rate of 14.1%, confirming the validity of the screening criteria published by Sperry et al.^18^ However, our value analysis shows that there are no cost savings per additional life year with the smallest ICER comparing NYHA class III to class IV of $75,333.28 USD/QALY. Although not all patients were candidates for tafamidis in our study, one patient is undergoing treatment to slow disease progression.

Tafamadis therapy has documented benefits to both morbidity and mortality when taken in a continuous fashion.^6^^,^^11^ In addition, tafamadis therapy has been shown to prolong the period during which patients remain in earlier stages of heart failure, thereby increasing the QALY gained.^5^^,^^6^^,^^11^ In this study, 84% of patients had no prior symptoms of heart failure at the time of biopsy. Even the remaining 16% of patients who had a history of heart failure NYHA class I or II at the time of CTR and biopsy. Therefore, a clear quality of life benefit is available for these patients diagnosed early in the disease course because of the disease-slowing effects of tafamadis therapy.

In our pilot study, cardiac work-up consisted of cardiac MRI, cardiac single photon emission computed tomography scan, cardiac nuclear medicine scintigraphy, echocardiogram, and serum monoclonal protein screening. This work-up pathway agrees with prior evaluation pathways described in the literature.^18^^,^^30^ Collectively, these prior studies support the possibility of the referral pathway outlined in this pilot study to detect amyloidosis in otherwise asymptomatic patients.^14^^,^^18^^,^^30^ With regards to the value component of this study, ICERs have been used widely in cost-utility analysis health economics to compare both the cost and effectiveness of two different treatments.^31^^,^^32^ When used in this type of analysis, a smaller ICER denotes less cost required for the gain of an additional QALY. These comparative measures are well suited for comparing the treatment of different groups of amyloid patients.

Our results show that there is no cost savings associated with early diagnosis of transthyretin amyloidosis and treatment in the earlier stages of heart failure. In addition, it is not cost effective to biopsy every patient who screens positive according to the criteria originally outlined by Sperry et al.^18^ Despite no projected costs savings with an earlier diagnosis, our reported ICER values are still an improvement upon a previously reported ICER of tafamadis treatment of 880,000/QALY.^31^ However, despite this improvement, when using a willingness to pay threshold of $50,000/QALY, early initiation of tafamadis at any stage does not represent a cost effective treatment.^28^^,^^29^ It is the opinion of the authors that an open dialog between cardiologists and orthopedic surgeons should be established to better identify high risk features and establish the optimal point in the disease course to begin treatment to simultaneously maximize benefit to patients who would clinically benefit from early intervention while minimizing the economic burden on the health system.

This study is not without limitations. For the cost of Congo red staining, we used hospital charge data from our institution which did not include charges associated with the physical collection of the biopsy specimen. However, given that the cost of Congo red staining is a small component of our ICER calculation, it is unlikely to significantly affect our cost calculation. This study investigated the use of tafamidis, which is only approved for the treatment of ATTR, as such conclusions from this study can only be applied to this subtype of amyloidosis. Furthermore, when determining the cost associated with hospitalizations because of heart failure, the referenced study by Parissis et al^23^ excluded patients who required intensive care unit (ICU) level of care as well as those who required vascular interventions while hospitalized. Given that the cost of ICU care is higher than typical inpatient care, our calculated cost because of hospitalizations is likely to be an underestimation of the true costs.

A final limitation of our study is the assumption of patients presenting in NYHA class I at the time of biopsy. NYHA class I heart failure is defined as patients with documented heart disease, eg, structural heart abnormalities or reduced ejection fraction who are asymptomatic both at rest and during ordinary physical activity.^24^^,^^33^ Given that carpal tunnel symptoms often predate cardiac symptoms by several years, the patients biopsied in this study are likely to be pre-class I NYHA.^34^ A hypothetical ICER equation for class 0 heart failure was generated to try and address this issue. The computed ICER comparison of class 0 to class IV was $202,417.75, which is a greater cost than comparing NYHA class I to NYHA class IV. This discrepancy can be accounted for by the length of time patients will be taking tafamadis therapy. Patients with class 0 heart failure are assumed to remain on tafamadis therapy for the remainder of their life. The increased cost per QALY gained is secondary to a longer life expectancy compared to those patients diagnosed in later stages of heart failure.

In conclusion, the results of this study demonstrate a potential improvement in the ICER value for tafamadis therapy secondary to early treatment initiation. However, the overwhelming costs of tafamidis treatment make the value of routine biopsy low. In line with the previous literature, for tafamidis to be considered a cost effective treatment a significant reduction in price would be required.^31^ Future investigation into determining which subset of factors place a patient at high risk for development of ATTR amyloidosis prior to cardiac involvement, as well as investigation into the optimal point in the disease course to begin tafamadis therapy are needed to increase the value of screening biopsy in patients undergoing routine CTR.

## Conflicts of Interest

Dr Day has received financial compensation from Arthrex and AM Surgical for research publications not related to this study. No benefits in any form have been received or will be received by the other authors related directly to this article.
